# CRISPR-Cas technologies: Emerging tools from research to clinical application

**DOI:** 10.71150/jm.2504012

**Published:** 2025-08-31

**Authors:** Hana Hyeon, Soonhye Hwang, Yongyang Luo, Eunkyoung Shin, Ji-Hyun Yeom, Hong-Man Kim, Minkyung Ryu, Kangseok Lee

**Affiliations:** 1 Department of Life Science, Chung-Ang University, Seoul 06974, Republic of Korea; 2 Department of Microbiology, School of Medicine, Catholic University of Daegu, Daegu 42472, Republic of Korea; 3 R & D Institute, NES Biotechnology, Seoul 06974, Republic of Korea

**Keywords:** CRISPR-Cas system, genetic engineering, clinical trial, delivery system, gene therapy

## Abstract

CRISPR-Cas technologies have emerged as powerful and versatile tools in gene therapy. In addition to the widely used SpCas9 system, alternative platforms including modified amino acid sequences, size-optimized variants, and other Cas enzymes from diverse bacterial species have been developed to apply this technology in various genetic contexts. In addition, base editors and prime editors for precise gene editing, the Cas13 system targeting RNA, and CRISPRa/i systems have enabled diverse and adaptable approaches for genome and RNA editing, as well as for regulating gene expression. Typically, CRISPR-Cas components are transported to the target in the form of DNA, RNA, or ribonucleoprotein complexes using various delivery methods, such as electroporation, adeno-associated viruses, and lipid nanoparticles. To amplify therapeutic efficiency, continued developments in targeted delivery technologies are required, with increased safety and stability of therapeutic biomolecules. CRISPR-based therapeutics hold an inexhaustible potential for the treatment of many diseases, including rare congenital diseases, by making permanent corrections at the genomic DNA level. In this review, we present various CRISPR-based tools, their delivery systems, and clinical progress in the CRISPR-Cas technology, highlighting its innovative prospects for gene therapy.

## The CRISPR-Cas Technology

The discovery of clustered regularly interspaced short palindromic repeats (CRISPR) in 1987 (Ishino et al., 1987) led to the identification of the CRISPR and CRISPR-associated protein (Cas) system as a defense mechanism in prokaryotes against phages (Barrangou et al., 2007; Garneau et al., 2010). In 2013, a pioneering study introduced the use of CRISPR-Cas9 for gene editing in prokaryotes (Jiang et al., 2013), and the same year, it was successfully used to edit the genome of eukaryotic cells (Cong et al., 2013).

CRISPR-Cas systems offer a range of features that support their therapeutic potential. The CRISPR-Cas system provides a simple and efficient gene editing process, enabling simultaneous targeting of multiple genes. In contrast to zinc-finger nucleases (ZFNs) (Kim et al., 1996) and transcription activator-like effector nucleases (TALENs) (Boch et al., 2009; Christian et al., 2010), CRISPR-Cas9 does not require a complex protein design for each target (Jinek et al., 2012; Ran et al., 2013). Moreover, this system is capable of manipulating the genomic sequences of cells and organisms, by enabling precise, locus-specific genome editing, they allow permanent correction of disease-causing mutations, in contrast to conventional drugs that often require lifelong administration and offer only transient effects. Additionally, CRISPR-Cas platforms are highly versatile, adaptable for gene disruption, correction, or epigenetic modulation. Their modularity and relative ease of design also facilitate rapid development across both ex vivo and in vivo therapeutic contexts, as well as introducing precise epigenetic and transcriptional modifications (Nakamura et al., 2021; Nishida et al., 2016).

## Cas Nuclease-Mediated Genome Editing

**Fig. 1.**
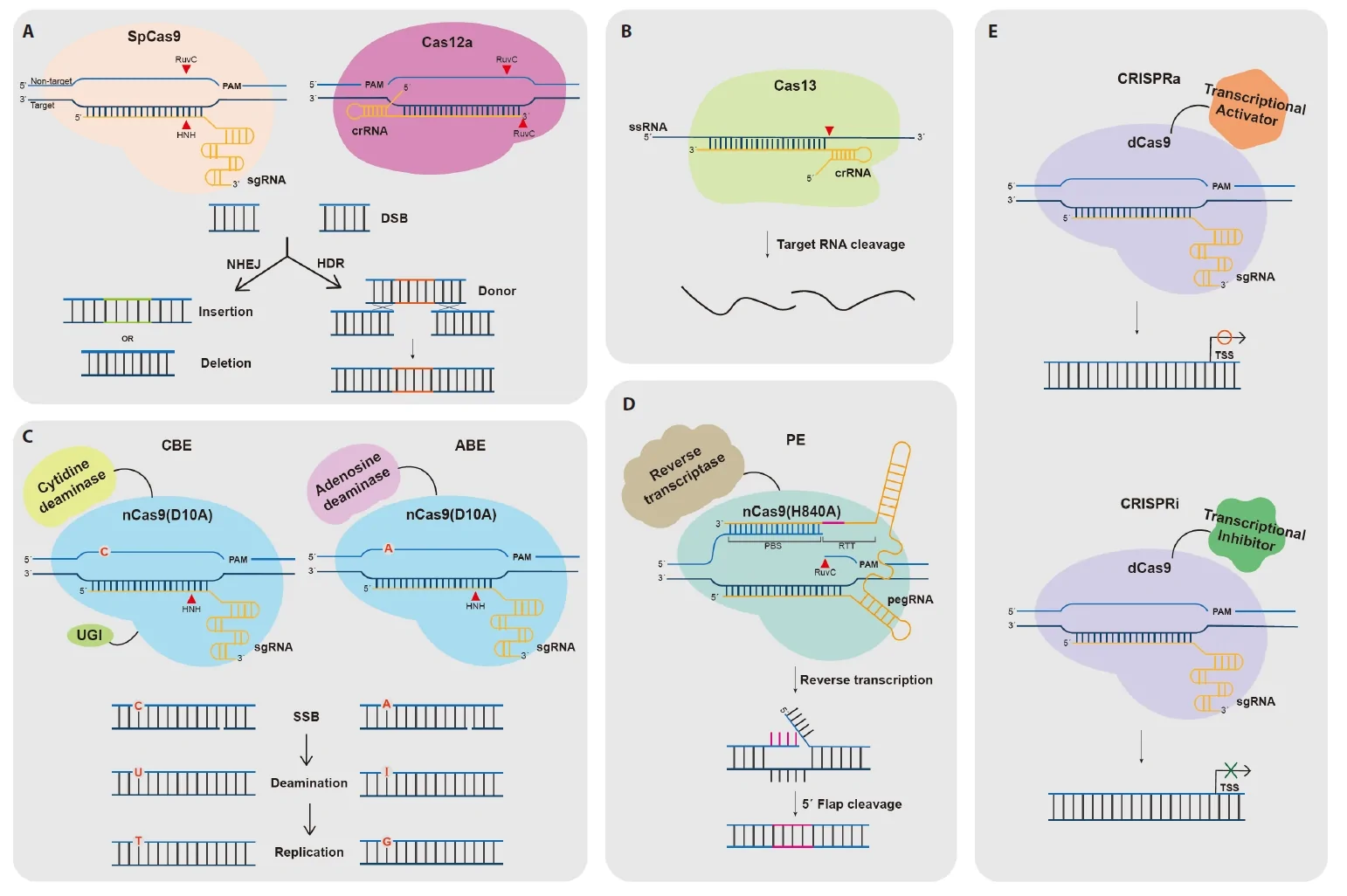
Diversity of CRISPR-based tools. Schematic diagrams of CRISPR-based tools and their mechanisms of action. (A) SpCas9 and Cas12a (Cpf1) nucleases generate DSB at target DNA sites guided by gRNAs, leading to indels via NHEJ or HDR pathways. (B) Cas13 cleaves target RNA in a crRNA-guided manner. (C) The base editing system utilizes deaminase enzyme fused to nCas9 to induce precise base substitutions without introducing DSBs. (D) The prime editing system employs nCas9 fused to a reverse transcriptase, guided by a pegRNA which also serves as template for targeted DNA synthesis. (E) The CRISPR activation/interference system involves dCas9 fused to transcription activator or repressor to epigenetically modulate gene expression without altering DNA sequences.

The CRISPR-Cas9 system is a highly effective genome editing tool that utilizes the endonuclease protein Cas9 and a guide RNA (gRNA) (Ran et al., 2013). The gRNA can be formed either as a duplex of CRISPR RNA (crRNA) and trans-activating CRISPR RNA (tracrRNA), or as a combined version, the single-guide RNA (sgRNA). The spacer region of the crRNA or 5′-end of the sgRNA forms complementary base pairing with the target sequence and guides Cas9 to it (Jinek et al., 2012). The specificity of target recognition and cleavage is ensured by the presence of the protospacer adjacent motif (PAM) (Anders et al., 2014). Upon target recognition and binding, the nuclease domains of Cas9, HNH and RuvC (Gasiunas et al., 2012), induce a double-strand break (DSB) by cleaving the target and nontarget DNA strands, respectively (Nishimasu et al., 2014) (Fig. 1A).

The DSB can be repaired via two pathways: non-homologous end joining (NHEJ) or homology-directed repair (HDR) (Ceccaldi et al., 2016; Sander and Joung, 2014). The NHEJ pathway often generates insertions or deletions (indels) that may cause frameshift mutations and premature stop codons, which can be used to reduce the expression of genes associated with various diseases (Frangoul et al., 2021; Gillmore et al., 2021). HDR is less frequent than NHEJ and requires the presence of donor DNA, but it can accurately introduce desired knock-in sequences at the target locus (Miyaoka et al., 2016; Yang et al., 2013a). Accordingly, clinical trials are currently investigating the application of HDR to correct disease-causing mutations through precise gene deletions or inversions (Kanter et al., 2021; Mark Walters, 2024).

The most well-studied and widely used Cas nuclease in type II CRISPR systems is SpCas9, which originated from *Streptococcus pyogenes* (Jiang and Doudna, 2017; Mali et al., 2013). Although it can accurately recognize PAM sequences and process its target DNA, its utility is limited to targeting DNAs with 5′-NGG-3′ PAM sequences (Jiang et al., 2013; Jinek et al., 2012). To address this inherent limitation, various Cas9 mutants that target altered PAM sequences by modifying the PAM-interacting (PI) domain have been developed. For instance, the PI domain of SpCas9 was modified to create variants that recognize the 5′-NGAN-3′, 5′-NGNG-3′, and 5′-NGCG-3′ PAM (Kleinstiver et al., 2015). Furthermore, an extended PAM variant, xCas9 has been developed to recognize multiple PAM sequences (5′-NG-3′, 5′-GAA-3′, 5′-GAT-3′, etc.) (Hu et al., 2018). Other SpCas9 variants have also been characterized; including a variant that recognizes a less restrictive 5′-NG-3′ PAM sequence (Nishimasu et al., 2018), as well as variants for non-G PAMs, such as 5′-NRRH-3′, 5′-NRCH-3′, and 5′-NRTH-3′ PAMs (Miller et al., 2020). Various Cas9 orthologs from diverse bacterial species that have different PAM specificities can also be used. These include SaCas9 (5′-NNGRRT-3′, R = A or G) (Kleinstiver et al., 2015; Ran et al., 2015), StCas9 (5′-NNAGAAW-3′, W = A or T) (Glemzaite et al., 2015; Muller et al., 2016), NmCas9 (5′-NNNNGMTT-3′, M = A or C) (Hou et al., 2013; Lee et al., 2016), CjCas9 (5′-NNNNRYAC-3′, Y = C or T) (Fonfara et al., 2014; Kim et al., 2017), and BlatCas9 (5′-NNNNCNAA-3′) (Gao et al., 2020; Karvelis et al., 2015).

Cpf1 (also known as Cas12a) has also been studied for its relative advantages, including high editing efficiency, small protein size, and dual functionality for both DNA and RNA cleavage. As a type V CRISPR, Cpf1 uses crRNA alone and induces staggered DNA DSBs with 4- or 5-nucleotide (nt)-long 5′ overhangs through a single RuvC-like domain (Zetsche et al., 2015) (Fig. 1A). AsCpf1 and LbCpf1, which can efficiently cleave target DNA regions with a short adenine-thymine (AT)-rich PAM (5′-TTTV-3′; V = A, G, or C) (Dong et al., 2016; Stella et al., 2017; Zetsche et al., 2015), expand the range of available PAM sequences.

Besides DNA, CRISPR-Cas systems can modify RNA. The CRISPR-Cas13 system, part of the type VI CRISPR system, features a single RNA-guided Cas13 protein with higher eukaryotes and prokaryotes nucleotide-binding (HEPN) and ribonuclease domains. This enables it to bind to and specifically cleave target single-stranded RNA through its ribonuclease activity (Abudayyeh et al., 2017) (Fig. 1B). The Cas13 family is classified into different subtypes based on the structural characteristics of their crRNA and the protein components, such as Cas13a (also known as C2c2), Cas13b, Cas13c, Cas13d, Cas13X, and Cas13Y (Liu and Pei, 2022). Cas13-based RNA editing systems have been effectively used for virus detection, splicing regulation, transcript labeling, and RNA knockdown (Cox et al., 2017; Freije et al., 2019; Konermann et al., 2018; Yang et al., 2019). Cas13-mediated RNA therapy offers a key advantage as it avoids irreversible genome mutations, that leads to RNA editing to gain popularity for treating diseases characterized by temporal changes in cellular states (Abudayyeh et al., 2019), particularly in the treatment of rare diseases (Cox et al., 2017; Tang et al., 2021).

For practical applications, the use of these nucleases in repairing intractable pathogenic mutations has been explored (Miller et al., 2020; Nishimasu et al., 2018). However, Cas variants exhibit notable limitations, including reduced efficiency and cleavage activity (Hu et al., 2018; Nishimasu et al., 2018), highlighting the need for further improvements to expand the application of Cas endonucleases in disease-relevant genome editing.

In addition to therapeutic applications, CRISPR-Cas systems are becoming a valuable tool for molecular diagnostics because of their programmability and high specificity. Certain Cas proteins (e.g., Cas12a, Cas13a) are used for signal amplification in diagnostic platforms that exploit target-dependent collateral cleavage activity; other Cas variants are catalytically inactive (e.g., dCas9), and they can be used to sequence-specific binding for sensor-based detection. These mechanistic features have led to the development of a range of diagnostic technologies (Kulkarni et al., 2023).

Early platforms such as Cas13a-based SHERLOCK (Kellner et al., 2019) and Cas12a-based DETECTR (Chen et al., 2018) demonstrated high sensitivity in detecting viral and bacterial pathogens. Subsequent innovations include FLASH-NGS (Quan et al., 2019), which utilizes Cas9 for targeted enrichment in sequencing applications; CASLFA (Wang et al., 2020a), combining Cas9 with lateral flow assays for rapid field diagnostics; CRISPR-Chip (Hajian et al., 2019), integrating dCas9 with graphene-based sensors for amplification-free detection; FELUDA (Azhar et al., 2021), employing FnCas9 for single nucleotide variant identification; VaNGuard (Ooi et al., 2021), designed for variant discrimination in SARS-CoV-2 detection; and CONAN (Shi et al., 2021), a Cas12a-based autocatalytic system achieving attomolar sensitivity without preamplification. These advancements highlight the versatility of CRISPR-Cas technologies in diagnostic applications.

## Base Editing

Besides leveraging the original function of the CRISPR-Cas9 system, its ability to localize proteins to specific target DNA sites via guide RNA has also been utilized. Variants, such as Cas9 nickase (nCas9) and catalytically inactive “dead” Cas9 (dCas9) (Qi et al., 2013), which were created by introducing mutations in one or both of the two catalytic domains of Cas9, have been used in conjunction with other enzymes to direct and mediate their function at the target site.

Base editors (BEs) are fusion proteins composed of nCas9 or dCas9 with a deaminase that promotes site-directed mutagenesis by single-base conversions at specific genomic locations targeted by sgRNA without generating DSBs (Gaudelli et al., 2017; Komor et al., 2016) (Fig. 1C).

Cytosine base editors (CBEs), such as APOBEC, consist of a fusion of either nCas9 (D10A) or dCas9 (D10A and H840A) and cytidine deaminase. An editor directed to a specific genomic locus by sgRNA can convert cytidine to uridine within a limited editing window adjacent to the PAM site. Subsequently, uridine is converted to thymidine via the base excision repair (BER) mechanism, resulting in a C-to-T transition (or a G-to-A transition on the complementary strand) (Komor et al., 2016). Adenine base editors (ABEs) utilize adenosine deaminase, a product of direct evolution of an *Escherichia coli* tRNA adenosine deaminase known as TadA. They are designed to convert adenosine to inosine, which is recognized as guanosine by DNA polymerase in the cell, resulting in an A-to-G (or a T-to-C) substitution (Gaudelli et al., 2017).

Additionally, two representative RNA BE systems were developed by fusing catalytically inactive Cas13 (dCas13) with the adenosine or cytidine deaminase domain. The RNA Editing for Programmable A-to-I Replacement (REPAIR) system induces adenosine-to-inosine deamination through the fusion of ADAR2 adenosine deaminase (Cox et al., 2017). Similarly, the RNA Editing for Specific C-to-U Exchange (RESCUE) system enables cytosine-to-uracil deamination by incorporating a directionally evolved ADAR2 cytidine deaminase (Abudayyeh et al., 2019).

Further advances have been made to improve the applicability of BEs. For instance, the PI domain mutants of nCas9 have been utilized to expand the target site, the linker region between the Cas variant and deaminase has been modified to adjust the editing window, and additional fusion proteins have been incorporated to increase the integrity of base conversion. Uracil DNA glycosylase inhibitor (UGI) was used to prevent the transformation of U into an apurinic/apyrimidinic site (Banno et al., 2018). In another study, bacteriophage Mu-derived Gam proteins were fused to Cas proteins to minimize the formation of unwanted indels that may be caused by DSBs during BER, utilizing the ability of Gam to bind to free DSB ends (Komor et al., 2017). In addition, new BEs with fewer PAM sequence constraints (Walton et al., 2020), C-to-G conversion capabilities (glycosylase base editors, GBEs) (Kurt et al., 2021; Zhao et al., 2021), and diverse editing windows (Koblan et al., 2018; Richter et al., 2020) have been developed.

As a therapeutic strategy, BEs have been applied to correct point mutations, such as pathogenic premature stop codons (Ryu et al., 2018), and to induce exon skipping by disrupting splice acceptors (CRISPR-SKIP) (Gapinske et al., 2018). By enabling precise editing to silence pathogenic mutant alleles or restore protein function, BEs hold a significant potential for managing a broad range of genetic disorders (Kuscu et al., 2017; Rossidis et al., 2018; Ryu et al., 2018). For instance, sickle cell anemia is caused by an A-to-T substitution in the β-globin gene (Kato et al., 2018). Other examples include cystic fibrosis (Amistadi et al., 2023) and phenylketonuria (Brooks et al., 2024). Although BEs are designed to avoid double-stranded DNA breaks, they can still lead to unintended indels and mutations. Previous research has shown that CBEs induce significant off-target single-nucleotide variants (SNVs) in both plant and animal models (Jin et al., 2019; Zuo et al., 2019). Additionally, there are limitations to this technology, such as in the correction of multi-nucleotide changes or small and large deletions. To overcome these limitations, new types of genome-editing tools, such as prime editors, have been developed (Anzalone et al., 2019).

## Prime Editing

The prime editor (PE) system is composed of an nCas9 (H840A)-reverse transcriptase (RT) fusion protein and a prime editing gRNA (pegRNA). PE does not require donor DNA templates or DSBs to generate insertions (up to 44 bp), deletions, or point mutations, such as transitions and transversions. Instead, the pegRNA contains a spacer sequence at its 5′-end and an extended 3′-end sequence that serves as both a primer binding site (PBS) and a reverse transcription template (RTT). The pegRNA guides nCas9 to its target DNA where it introduces a single-stranded break exposing a 3′ DNA end. The PBS in the pegRNA anneals to this region to allow the RT to begin DNA synthesis using the RTT, which results in a 3′ DNA flap with the intended edit. The newly synthesized flap is resolved by cellular repair mechanisms and integrated into the genome resulting in stable genome modification (Anzalone et al., 2019). Large genomic deletions or insertions of up to 110 bp can be generated using a pair of partially complementary pegRNAs (paired-peg, TWIN-PE) (Anzalone et al., 2022) (Fig. 1D).

The PE concept holds promise for precise and potentially limitless genome editing, which can greatly expand its applications in biological and medical research. However, it faces key shortcomings that require further optimization. First, the editing efficiency of PEs is lower than that of BEs, which may limit their therapeutic utility. Furthermore, the large size of the fusion protein makes it difficult to load onto delivery materials. Thus, the development of strategies for more efficient delivery is required. Therefore, these shortcomings must be overcome to realize the full therapeutic potential of PEs. If prime editing continues to improve, theoretically approximately 89% of known disease-causing gene mutations can be corrected (Anzalone et al., 2019).

## CRISPRa/i for Transcriptional Regulation

In addition to modifying bases and deleting or integrating DNA sequences, the CRISPR-Cas9 system can be used to manipulate gene expression by combining transcription factors (TFs) and can function as transcriptional repressors (CRISPR interference; CRISPRi) or activators (CRISPR activation; CRISPRa) (Gilbert et al., 2014) (Fig. 1E). The CRISPRi system utilizes repressor domains, such as the Krüppel-associated box (KRAB) (Margolin et al., 1994) to create steric hindrance that blocks the binding of TFs to the promoter region, resulting in decreased gene expression (Gilbert et al., 2013). Conversely, the CRISPRa system integrates activator domains, such as VP64, Rta, HSF1, and p65, to recruit RNA polymerase or other TFs, thereby enhancing the expression of downstream genes (Chavez et al., 2015; Nihongaki et al., 2019). These systems are promising for therapeutic use because they can function without permanent genomic modifications. Although these tools have fewer clinical applications, recent in vivo studies have shown that CRISPRa and CRISPRi approaches are therapeutically feasible for a variety of disease models (e.g., CRISPRi to repress the *Nrl* gene in retinitis pigmentosa model mice to prevent secondary cone loss [Moreno et al., 2018], downregulating *Pcsk9* expression in the mouse liver to reduce cholesterol serum level [Thakore et al., 2018], silencing *Fabp4* in white adipose tissue of obesity-induced diabetic mouse model for metabolic homeostasis [Chung et al., 2019], suppressing oncogenic Δ*Np63* in xenograft mouse model to inhibit tumor growth [Yoshida et al., 2018]), and CRISPRa to activate *Fgf21* expression in hepatic tissue to improve metabolic regulation in adult mice [Zhang et al., 2021]). Therefore, sustained expression of the CRISPRa/i system is required to ensure stable transcriptional regulation during therapeutic interventions.

## CRISPR-Cas Delivery Formats and Tools

The CRISPR-Cas system offers a precise and direct approach to treating genetic disorders, making it an ideal candidate for therapeutic applications. For the safe and effective clinical implementation of CRISPR-Cas, the development of suitable format of cargo and delivery systems for both in vivo and ex vivo applications is imperative (Table 1).

## CRISPR-Cas Cargo Formats

The CRISPR-Cas9 system is available in various formats, including 1) a plasmid encoding both Cas9 and sgRNA (Sakuma et al., 2014); 2) two separate plasmids, which encode Cas9 and sgRNA, respectively (Senís et al., 2014); 3) Cas9 mRNA together with the sgRNA molecule (Miller et al., 2017); and 4) ribonucleoprotein (RNP) complexes comprising the Cas9 endonuclease protein and the sgRNA molecule (Schumann et al., 2015). Each format offers different advantages and limitations depending on the specific application and delivery method (Salvagnin et al., 2023; Yin et al., 2016).

Delivering the CRISPR-Cas system in the plasmid DNA form allows for sustained expression of Cas and gRNA, which can be advantageous in cases requiring continuous gene editing. Plasmids are attractive options for laboratory and therapeutic applications because of their high stability, cost-effectiveness, and scalability for large-scale manufacturing (Slattery et al., 2018; Zhang et al., 2020). However, plasmid delivery also has several disadvantages. Owing to its complex structure and additional genetic elements, such as promoters and antibiotic resistance genes, plasmid DNA is generally larger than mRNA and RNP formats (Lin et al., 2022). Additionally, because the Cas9 protein requires to be transcribed in the nucleus, the plasmid must cross both the plasma and nuclear membranes for successful delivery, posing significant challenges, especially in eukaryotic cells (Glass et al., 2018; Vaughan and Dean, 2006). Furthermore, random integration of the plasmid into the host genome may induce sustained expression of the Cas9 protein, which can lead to off-target gene editing and potentially trigger an immune response to foreign DNA (Cho et al., 2014; Pattanayak et al., 2013).

Another option, the mRNA format, is translated directly in the cytoplasm, which eliminates the need for nuclear entry and transcription and allows for a faster onset of gene editing. Owing to the unstable nature of RNA, transient expression of the Cas9 protein also reduces off-target effects and the risk of genome integration (Leonhardt et al., 2014). However, producing Cas9 and gRNA in an mRNA format is generally more expensive and difficult because of the large size of the expression cassettes. Additionally, mRNA exhibits lower stability than DNA in biological fluids and is highly sensitive to temperature fluctuations, necessitating the use of low-temperature storage systems (Uddin and Roni, 2021; Zhang et al., 2020). Finally, the RNP format, which includes the Cas9 protein and gRNA complex, does not require transcription or translation. This format provides for the fastest-acting approach, yielding high editing efficiencies, and minimizing the risk of off-target side effects owing to its short duration of activity (Kouranova et al., 2016). However, because of the complex composition and charge properties of RNPs, lipid nanoparticle (LNP) and adeno-associated virus (AAV) systems, which have been extensively studied and are commonly used for DNA or RNA delivery, are not readily applicable for RNP delivery (Chen et al., 2019; Wei et al., 2020).

## CRISPR-Cas Delivery Systems

Currently, CRISPR-Cas components are delivered via three main delivery platforms: physical methods, viral vectors, and nonviral vectors. Physical methods, such as electroporation and microinjection, facilitate the direct delivery of therapeutic molecules into cells by inducing the formation of temporary nanopores on membranes or by directly introducing the materials (Tsong, 1991). Electroporation can achieve high delivery efficiency with a variety of CRISPR-Cas formats, including DNA, mRNA, and RNPs, owing to its mode of action (Kang et al., 2015; Ren et al., 2017; Schumann et al., 2015). This method bypasses the need for a carrier and imposes few restrictions on the cargo size, typically found with viral vectors (Atkinson and Chalmers, 2010), and unlike nonviral vectors, it is not constrained by endocytosis as a rate-limiting step (Fajrial et al., 2020). In vivo delivery of plasmid-based CRISPR-Cas9 components via electroporation has been successfully applied in preclinical models. For example, CRISPR-Cas9 plasmids targeting *Dmd* were delivered into the skeletal muscle of *mdx* mice, resulting in restored dystrophin expression and improved muscle function (Xu et al., 2016). In another study, co-delivery of Cas9 RNP and donor DNA into retinal pigment epithelial cells rescued photoreceptor degeneration in a model of retinitis pigmentosa (Cai et al., 2019). However, electroporation is generally limited to ex vivo administration because of the challenges involved, such as the possible impairment of cell viability by strong electric fields (Bak et al., 2017; Canatella et al., 2001). The use of 100-volt pulses during electroporation for drug delivery to the skin causes a painful sensation and can lead to injury (Prausnitz, 1999; Weaver et al., 1997). Therefore, the delivery conditions using electroporation must be carefully adjusted based on the cell type and other experimental parameters to achieve optimal performance.

Viral vectors, including lentivirus vectors (LVs), adenovirus vectors (AdVs), and adeno-associated virus vectors (AAVs), use the viral machinery to deliver therapeutic agents (Dong and Kantor, 2021; Tsukamoto et al., 2018; Verdera et al., 2020). Despite the clinical advantages of viral vector-based gene therapy, all suffer from specific limitations that limit more widespread application. Lentiviral vectors have the advantage of stably integrating transgenes, but they also run the risk of insertional mutagenesis (Ranzani et al., 2013) and are difficult to production on a large scale (Valkama et al., 2020). Adenoviral vectors allow high payloads of genes to be delivered, but they elicit strong innate and adaptive immune responses (Lowenstein et al., 2007; Zhu et al., 2007) that limit their repeat use and safety, due primarily to concerns about vector recombination (Walsh et al., 2009).

Currently, AAVs are the preferred vectors for in vivo gene delivery with some advantages over other viral vectors, including limited integration into the host genome and relatively low immunogenicity (VandenDriessche et al., 2007). Various AAV serotypes exhibit specific tissue tropism, notably for lung epithelial cells, cardiac cells, neurons, and skeletal muscle cells, enabling targeted gene delivery to specific tissues (Aschauer et al., 2013; Bish et al., 2008; Blankinship et al., 2004; Halbert et al., 2001).

AAVs are highly prevalent in humans, with approximately 80% of the population exhibiting seropositivity for at least one AAV serotype. Despite their extensive distribution, no human disease has been linked to AAV infections (Chhabra et al., 2024; Flotte et al., 2022; Lek et al., 2023b). Owing to their favorable safety profile, AAVs have become a leading viral delivery system for in vivo delivery of CRISPR-Cas components (Kim et al., 2017; Wang et al., 2020b).

Nonetheless, AAVs have critical limitations such as pre-existing neutralizing antibodies (Mendell et al., 2022) and difficulty with high-titer production. Particularly, AAVs have a critical limitation in terms of their DNA packaging capacity, which is approximately 4.7–5 kb (Dong et al., 1996). This limitation poses a challenge for the use of larger CRISPR-Cas systems. The SpCas9 protein alone is encoded by DNA that is more than 4.1 kb in size, and when combined with sgRNA sequences and regulatory elements, the total payload frequently exceeds the capacity of a single AAV vector (Senís et al., 2014). Consequently, CRISPR-based gene editing using AAV vectors often requires multiple vectors, making the transfection process more complex, time-consuming, and costly. Strategies to overcome this limitation include the use of smaller Cas9 orthologs or Cas12f1 (Kim et al., 2022; Ran et al., 2015). Another approach involves the use of dual AAV systems that independently express Cas proteins and gRNAs using two distinct vectors (Yang et al., 2016). Additionally, split intein reconstitution is employed to deliver larger components, such as BEs and PEs, by assembling full-length proteins from split fragments after expression (Levy et al., 2020; She et al., 2023; Truong et al., 2015).

Nonviral vectors, such as LNPs, offer a safer alternative with lower immunogenicity, although their delivery efficiency is often lower than that of viral methods (Chen et al., 2017; Pérez-Martínez et al., 2011; Uchida et al., 2002), and also, unlike electroporation, LNPs are FDA-approved drug delivery systems that do not impose significant stress on cells (Adams et al., 2018). LNPs, including liposomes, are widely used as carriers for delivering various molecules, particularly nucleic acids, into cells (Hou et al., 2021; Kulkarni et al., 2019). The strong anionic charge of nucleic acids and their inherent instability outside cells make their passage across the negatively charged cell membrane extremely difficult. Thus, encapsulation in cationic liposomes enables efficient delivery by facilitating membrane fusion and intracellular release (Gao and Huang, 1996).

The delivery of CRISPR-Cas9 systems using LNPs can occur in the form of DNA (Zhang et al., 2017), mRNA (Yin et al., 2016), or RNPs (Zuris et al., 2015). For gene editing, CRISPR-Cas9 systems have been successfully delivered both in vitro and in vivo using the commonly employed Lipofectamine transfection (Schuh et al., 2018; Schwank et al., 2013; Yu et al., 2016). Lipid-based reagents for CRISPR-Cas9 delivery have been utilized in clinical trials for the treatment of various diseases, such as transthyretin amyloidosis, hereditary angioedema, and calcific aortic valve stenosis (Adams et al., 2018; Gillmore et al., 2021; Longhurst et al., 2024; Morrow et al., 2023).

Although LNPs have several advantages, they are typically sequestered within endosomes. To prevent lysosomal degradation of the cargo, LNPs must efficiently escape endosomes after crossing the cell membrane (Gilleron et al., 2013). Even though Cas9 complexes successfully escape the endosome, efficient nuclear transport remains a significant challenge, frequently leading to suboptimal delivery efficiency (Shen et al., 2013). Furthermore, LNP-based delivery is associated with dose-dependent toxicity and potential immunogenicity, which pose challenges to its therapeutic applicability (Kedmi et al., 2010; Swaminathan et al., 2016). As for targeted delivery, LNPs primarily accumulate in the liver, where lipid uptake and metabolism are mediated by the low-density lipoprotein receptor (LDLR). Therefore, modulation of the surface charge by recombining the components of LNPs is being focused upon to improve delivery to specific organs that require gene editing (Cheng et al., 2020; Wei et al., 2020).

The currently used delivery systems are limited in their inability to customize the delivery formats, duration of expression, and expression levels of CRISPR-Cas. To overcome these challenges, novel delivery platforms, such as AuNPs, are being explored. AuNPs are characterized by low toxicity, nonimmunogenicity, biocompatibility, and highly tunable surface chemistry (Carnovale et al., 2016; Shukla et al., 2005). Their favorable chemical properties facilitate simple conjugation methods, particularly via interactions with materials containing thiol groups (Cutler et al., 2012; Liu and Liu, 2017; Storhoff et al., 1998). This allows for the delivery of DNA, RNA, and proteins via a single platform through the utilization of pre-existing thiol groups or thiolation of the terminal or externally exposed regions of each molecule. Several studies have demonstrated successful in vitro and in vivo delivery of RNA (Yeom et al., 2013), single- or double-stranded oligonucleotides (Jensen et al., 2013; Kim et al., 2010, 2011; Ryou et al., 2010), peptides (Lee et al., 2017b; Yeom et al., 2016), proteins (Ryou et al., 2014), and antibodies (Yeom et al., 2023) using AuNPs. AuNPs are particularly advantageous for localized genome editing because they allow targeted delivery by simultaneously carrying both active substances and targeting molecules. Furthermore, AuNPs are safe for clinical use and have been approved by the FDA for clinical research (Kumthekar et al., 2021). Given their safety and versatility, AuNPs have also been explored as delivery vehicles for CRISPR-Cas in the RNP format. Successful gene editing has been demonstrated in both in vivo and in vitro models using this platform (Lee et al., 2017a, 2018; Shahbazi et al., 2019), which highlights the potential of AuNPs as future therapeutic platforms, subject to further research and development.

## CRISPR-Based Genetic Therapies in Clinical Trials

**Fig. 2.**
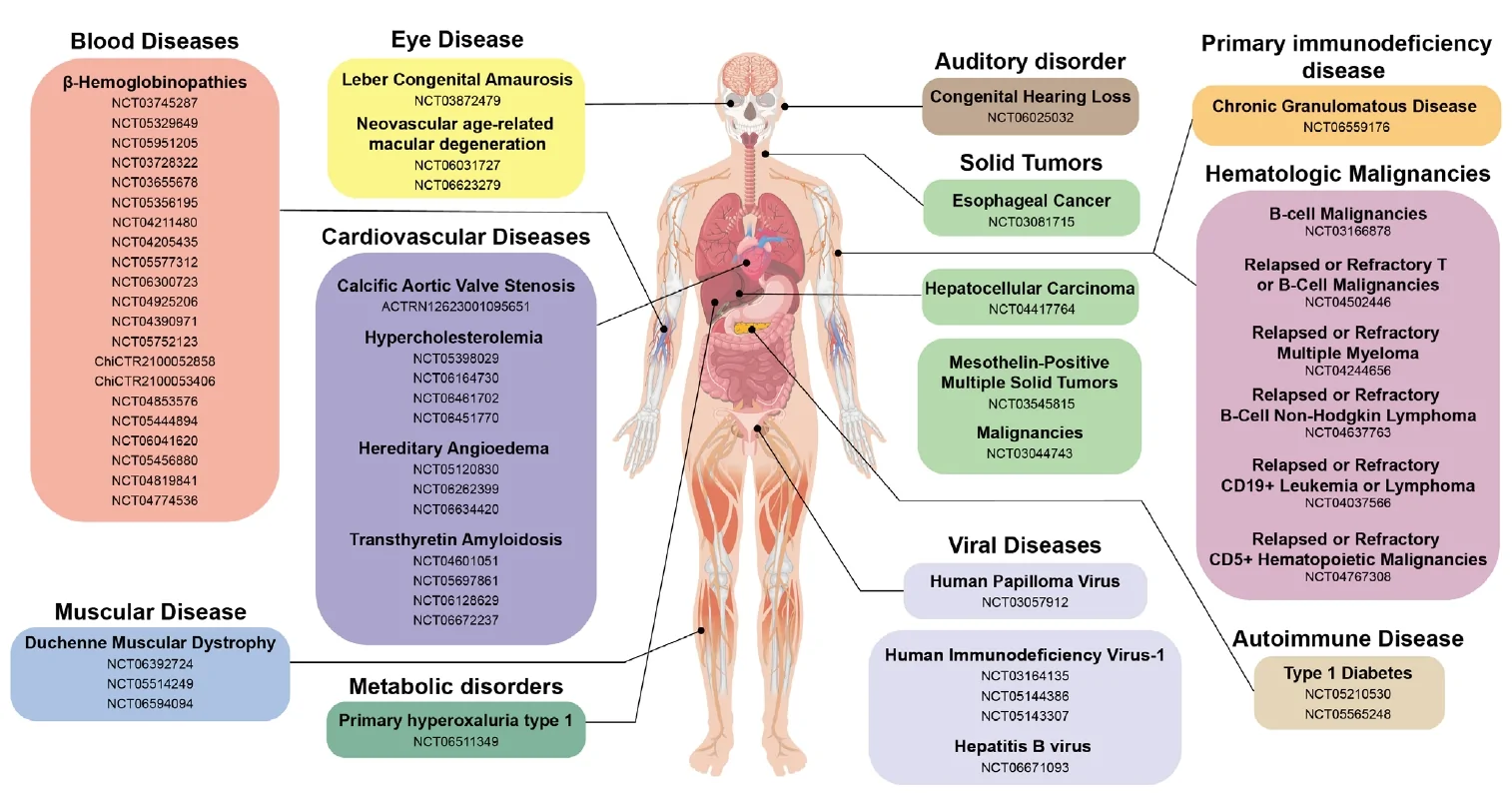
Clinical trials of CRISPR-based genetic therapies. Schematic illustration of human anatomical structures and associated diseases investigated in clinical studies using CRISPR-based tools. NCT numbers are classified for different diseases, including cardiovascular diseases, eye diseases, blood diseases, metabolic disorder, muscular disease, auditory disorder, solid tumors, viral diseases, primary immunodeficiency disease, autoimmune disease, and hematologic malignancies, according to the affected organs.

CRISPR-based gene editing has completely changed the scenario in the field of medical research, enabling the rapid development of cellular and animal models for preclinical and clinical development of novel treatment strategies. By directly altering the genome, CRISPR-Cas can offer long-term benefits to patients in contrast to traditional drug treatments that provide temporary relief. Furthermore, by easily changing the sgRNA sequence and adjusting the Cas9 variants, the system can be quickly implemented to provide a practical and effective therapeutic option for a variety of diseases. As described previously, the CRISPR-Cas system is a promising platform for gene editing. To highlight its usefulness, development of therapeutic approaches using the CRISPR-Cas system and related clinical trials of this technology are described in this section (Fig. 2 and Table 2).

## Blood Disease

β-Hemoglobinopathies, which affect the β-chain of hemoglobin, impair the efficiency of oxygen transport by hemoglobin. These disorders are among the most prevalent monogenic diseases worldwide, with sickle cell disease (SCD) and β-thalassemia being the predominant forms (Piel et al., 2013). SCD results from a 20A>T mutation in the β-globin gene (*HBB*), leading to the production of hemoglobin S (HbS, β^Glu6Val^) (Kato et al., 2018). The sickle-shaped red blood cells caused by the mutation induce hemolytic anemia and vaso-occlusive events, resulting in ischemic damage to tissues and contributing to acute pain crises and organ failure (Piel et al., 2017). In contrast, β-thalassemia is resulting from a variety of mutations, including deletions that reduce the synthesis of functional β-globin protein. This imbalance between β- and α-globin chains leads to the precipitation of α-globin within red blood cells, causing hemolysis and impaired erythropoiesis (Taher et al., 2021).

Many gene editing approaches for β-hemoglobinopathies focus on increase fetal hemoglobin (HbF, α2γ2) levels by damaging B-cell lymphoma/leukemia 11A gene (*BCL11A*), which suppresses γ-globin expression, and the promoter regions of the γ-globin genes (*HBG1/2*), ex vivo in HSPCs, to substitute γ-globin for β-globin (Forget, 1998; Li et al., 2018). In 2018, clinical trials were initiated to treat severe SCD (NCT03745287; NCT05329649; NCT05951205) (Vertex Pharmaceuticals, 2018b, 2022a, 2024) and transfusion-dependent β-thalassemia (TDT) (NCT03728322; NCT03655678; NCT05356195) (Allife Medical Science and Technology, 2019; Vertex Pharmaceuticals, 2018a, 2022b), by targeting the DNase I hypersensitivity sites (DHS) in erythroid-specific enhancer of *BCL11A* in autologous CD34^+^ cells ex vivo (Canver et al., 2015; Wu et al., 2019). Those led to the U.S. Food and Drug Administration (FDA) approval in 2023 of Exagamglogene autotemcel (Exa-cel; marketed as CASGEVY), the first CRISPR-Cas9-based ex vivo gene editing therapy. Preliminary findings demonstrated a high rate of successful incorporation of genetically modified HSPCs, with 80% of the alleles exhibiting modification of *BCL11A*, 1 year after the treatment. This led to a marked increase in HbF production, accompanied by a significant reduction in the need for blood transfusions as well as in the incidence of vaso-occlusive events in patients with SCD (Frangoul et al., 2021). To disrupt the +58 DHS of *BCL11A*, two similar approaches are currently under clinical evaluation for the treatment of TDT, developed by Bioray (BRL-101) (NCT04211480, NCT04205435, NCT05577312, and NCT06300723) (Bioray Laboratories, 2020, 2021, 2022, 2024) and EdiGene (ET-01) (NCT04925206, NCT04390971, and NCT05752123) (EdiGene, 2021, 2023; Institute of Hematology & Blood Diseases Hospital, 2023b) (Fang et al., 2019; Zheng et al., 2023). During an 18-month follow-up study of BRL-101, two patients demonstrated successful engraftment of modified HSPCs, resulting in an editing incidence of 85% in the bone marrow and a significant increase in HbF levels (Fu et al., 2022). These outcomes were also observed in 10 patients diagnosed with TDT (Zheng et al., 2023). Similarly, the initial findings from ET-01 exhibited encouraging results (Shi et al., 2022).

In parallel with this, HSPCs edited using the CRISPR-Cas9 to disrupt *BCL11A* binding site at *HBG1/2* (ChiCTR2100052858; ChiCTR2100053406) (The First Affiliated Hospital of Guangxi Medical University, 2021; The 923rd Hospital of the People's Liberation Army, 2021) showed increased HbF levels in individuals with TDT (Liu et al., 2023, 2024; Wang et al., 2022). Additionally, EDIT-301 employs an AsCas12a-based genome editing approach in HSPCs to effectively disrupt the repressor-binding sites of the *HBG1/2* promoter via electroporation (De Dreuzy et al., 2019; Hanna et al., 2023). This results in a 40% increase in HbF, and are currently undergoing clinical assessment for SCD (NCT04853576) (Editas Medicine, 2021) and TDT (NCT05444894, NCT06041620) (Editas Medicine, 2022; Institute of Hematology & Blood Diseases Hospital, 2023a). Beam Therapeutics developed BEAM-101, which introduces an ABE system to HSPCs by electroporation, to induce a point mutation in the regulatory element of *HBG1/2* promoters and reactivating γ-globin expression (NCT05456880) (Beam Therapeutics, 2022; Chockalingam et al., 2024; Gupta et al., 2024).

Alternative approach for treating SCD involves direct correction of *HBB* mutations. Clinical trials have attempted to induce HDR by delivering Cas9 RNP into HSPCs via electroporation, with donor DNA introduced through rAAV6 transduction (Dever et al., 2016; Lattanzi et al., 2021) (NCT04819841) (Kamau Therapeutics, 2021; Kanter et al., 2021) or electroporation (DeWitt et al., 2016) (NCT04774536) (Mark Walters, 2024). Although still in the preclinical stage, BEs hold a promising therapeutic approach. While those cannot directly reverse the A-to-T mutation, ABEs have enabled conversion of the *HBB^S^* (HbS; βE6V) allele to a naturally occurring nonpathogenic variant *HBB^G^* (HbG-Makassar; βE6A), restoring normal hemoglobin function without off-target effects (Chu et al., 2021; Newby et al., 2021).

## Muscular Disease

Duchenne muscular dystrophy (DMD) is the most common genetic muscular disorder in humans, especially in male (Mendell et al., 2012), that causes severe and progressive muscle weakness and wasting due to insufficient expression of dystrophin from *DMD* (Guiraud et al., 2015). *DMD* is the largest human gene (79 exons over 2.2 Mb of genomic DNA) located on the X-chromosome, and the site of numerous mutations, primarily deletions (68%), point mutations (11%), and duplications (11%) (Aartsma‐Rus et al., 2006; Bladen et al., 2015). Dystrophin is essential for preserving the biomechanical characteristics of fiber strength, flexibility, and stability in muscle (Blake et al., 2002). Through the dystrophin-associated protein complex (DAPC), the dystrophin protein functions as a molecular shock absorber, creating a mechanical connection between the extracellular matrix and actin cytoskeleton.

Most patients succumb to death in their early adult years due to heart and respiratory failure, with a median survival of 28.1 years (95% CI 25.1, 30.3) (Broomfield et al., 2021). Despite intensive clinical efforts focused on managing coronary heart disease, providing respiratory support, and administering corticosteroid, this debilitating illness remains incurable. Recent studies have explored the use of CRISPR-Cas, BEs, and CRISPRa technologies to restore dystrophin expression in affected patients. CRISPR-Cas is able to induce indels at the splice donor or acceptor site, and skipping exon 51 resulted in the production of a shorter but functional dystrophin protein that could improve muscle function (Amoasii et al., 2017). Under this strategy, a clinical trial (NCT06594094) (HuidaGene Therapeutics, 2024) is in progress using CRISPR-hfCas12Max (Zhang et al., 2022a), delivered via a single all-in-one AAV vector, intravenously. This therapeutic candidate, HG302, demonstrated restoration of dystrophin protein expression in muscle fibers and improvement of muscle function to near wild-type levels in preclinical studies involving humanized *DMD* mouse models (HuidaGene Therapeutics, 2023b). Similarly, another clinical trial (NCT06392724) (GenAssist, 2024) for GEN6050X (GenAssist) aims to skip exon 50 using its proprietary Targeted AID-mediated Mutagenesis (TAM) CBE, delivered intravenously via dual AAV9 vectors (Yuan et al., 2018).

The regulation of gene expression through CRISPRa facilitates the modulation of disease-modifying genes, potentially delaying disease progression or alleviating symptoms, thereby providing therapeutic opportunities for all patients with DMD regardless of their dystrophin mutations (Mollanoori et al., 2021). Recently, Cure Rare Disease, Inc. in the United States initiated the first CRISPR-based clinical trial on DMD (NCT05514249) (Cure Rare Disease, 2022). This n-of-1 clinical trial was conducted on a 27-year-old patient with DMD having muscular dystrophin deficiency due to an exon 1 deletion. The therapeutic agent named CRD-TMH-001 was designed to upregulate the expression of an alternative dystrophin isoform to bypass the effect of mutation by intravenously delivering a high dose of AAV9-dSaCas9-VP64 (1 × 10^14^ vg/kg), where VP64 is tetrameric repeat of the herpes simplex virus type 1 transcription activator VP16 (Perez-Pinera et al., 2013). Regrettably, the patient experienced cardiac arrest and died two days later, and autopsy findings indicate lung damage as the underlying cause. The study team concluded that these side effects were not due to the CRISPR-Cas technology itself, but rather due to the high-dose usage of AAV (Lek et al., 2023a). It highlights the toxicity concerns associated with AAV-based gene therapies and has important implications for the future development of gene therapeutics.

## Eye Disease

**Leber congenital amaurosis (LCA):** Leber congenital amaurosis (LCA) is an inherited degenerative retinal disease that results in severe visual impairment and blindness at an early age, with an incidence of approximately 1 in 80,000 (Tsang and Sharma, 2018). This disease has more than 20 types and is distinguished by genetic causes and symptoms. The most common type, LCA10, results from loss-of-function mutations in *CEP290*, which is essential for the assembly and phototransduction of the photoreceptor cilia(den Hollander et al., 2006; Stone, 2007). The IVS26 mutation (c.2991 + 1655A > G) in intron 26 of *CEP290* creates an aberrant splice site, resulting in a premature stop codon, and accounts for more than 15% of all LCA cases (den Hollander et al., 2006).

Recent advances in CRISPR-Cas9 gene editing, the removal or correction of the IVS26 mutation via NHEJ and HDR pathways in iPSC and LCA10 mouse models have demonstrated potential therapeutic effects (Burnight et al., 2017; Maeder et al., 2019; Ruan et al., 2017). EDIT-101, developed by Editas Medicine, is designed to remove IVS26 mutation through subretinal injection of an AAV5 vector delivering SaCas9 and two sgRNAs that target sequences flanking the mutation site, thereby restoring normal CEP290 expression (Ledford, 2020). Phase I/II clinical trial (NCT03872479) (Editas Medicine, 2019) established it as the first in vivo CRISPR-Cas9 gene-editing therapy to receive regulatory approval for clinical trial initiation, and the trial reported no severe adverse effects related to the treatment or procedure, and no dose-limiting toxicities were observed (Pierce et al., 2024).

**Neovascular age-related macular degeneration (nAMD):** Age-related macular degeneration (AMD) is the most common cause of blindness in the elderly. It is characterized by irreversible vision loss caused by a progressive deterioration of the macula, the central region of the retina (Ferris et al., 1984). Neovascular AMD (nAMD), which accounts for 80-90% of AMD blindness, is primarily caused by abnormalities in vascular endothelial growth factor (VEGF) signaling (Bressler et al., 1988). Overexpression of VEGF-A, one of several isoforms of VEGF, leads to abnormal growth of choroidal neovascularization (CNV), which is the key pathological characteristic of nAMD (Amadio et al., 2016). The result of neovascularization is very delicate and susceptible to bleeding and fluid leakage, leading to deterioration of central vision.

HuidaGene Therapeutics developed HG202 (NCT06031727, NCT06623279) (HuidaGene Therapeutics, 2023a, 2025), an RNA targeting gene therapy that utilizes high-fidelity CRISPR–Cas13Y delivered via unilateral subretinal injection using an rAAV vector to knock down VEGF-A expression (Tong et al., 2023). This strategy was demonstrated by a single subretinal treatment that suppressed *VegfA* mRNA expression by more than 40% and reduced the area of laser-induced CNV in the eyes by 87% in mice (Shi et al., 2023, 2024).

## Auditory Disorder

Congenital hearing loss refers to hearing impairment present before a child acquires speech abilities and affects approximately 1 in 500 newborns (Mehl and Thomson, 1998). One of the primary causes of auditory neuropathy spectrum disorder and the cause of 1–8% of congenital non-syndromic hearing loss is the c.2485 C > T (p.Q829X) nonsense mutation in the *OTOF*, which encodes the calcium-binding protein otoferlin (Iwasa et al., 2013; Migliosi et al., 2002; Yang et al., 2013b). HG205 was evaluated in clinical trials (NCT06025032) (HuidaGene Therapeutics, 2023c) as a treatment that restores functional protein expression by targeting mutant mRNA rather than altering genomic DNA. The approach involves delivering a CRISPR-Cas13 system via intracochlear injection of an AAV vector, enabling RNA base editing to repair the mutation at the transcript level (Xue et al., 2023), however, the trial was withdrawn due to the absence of enrolled patients in China.

## Autoimmune Disease: Type 1 Diabetes Mellitus (T1D)

T1D is an autoimmune disease characterized by the immune-mediated destruction of pancreatic β-cells, which are responsible for insulin production (Harrison et al., 2004). Insulin deficiency leads to chronic hyperglycemia, which can cause long-term complications affecting various organs, including the eyes, cardiovascular system, kidneys, nerves, and oral health (Shojaeian and Mehri-Ghahfarrokhi, 2018). Although T1D can occur at any age, it is more frequently identified during childhood or adolescence and resulted in life-threatening in the absence of appropriate treatment. The conventional treatment for T1Ds includes frequent blood glucose monitoring and subcutaneous insulin injections, requiring strict adherence to administration protocols to maintain blood glucose levels (Aathira and Jain, 2014). Because of the inconvenience of daily insulin injections, the transplantation of autologous stem cell-derived β-cells offers unlimited cell supply and avoids graft rejection for patients with T1D (Millman et al., 2016). However, without immunosuppression, persistent autoimmune responses rapidly destroy the transplanted cells and immunotherapy to enhance β-cell tolerance has not yet been successfully developed (Atkinson et al., 2019), limiting this approach to the most severe cases and hindering broader application (Bruni et al., 2014; Gruessner and Sutherland, 2005).

In the absence of a definitive cure, CRISPR-Cas-based therapeutic strategies suggest various approaches to improve β-cell survival by modulating immune responses, and increase insulin production. A representative method involves the CRISPR-Cas9-mediated knockout of β2-microglobulin (*B2M*), an essential component of the MHC-I signaling pathway, followed by insertion of the *PD-L1* at *B2M* locus by HDR pathway to facilitate circumvention of transplant rejection (Sluch et al., 2019). Based on this concept, in 2022, CRISPR Therapeutics and ViaCyte introduced VCTX210A. VCTX210A is composed of genetically modified allogeneic pancreatic endoderm cells (PEC210A) encapsulated within an implantable device. Following implantation, the cells differentiate into β-cells and other islet cells in the perforated device and started to supply insulin into the blood. The efficacy and safety of VCTX210A are being evaluated in clinical trial NCT05210530 (CRISPR Therapeutics, 2022; Philippidis, 2022). Additionally, a new study, NCT05565248 (CRISPR Therapeutics, 2023a), is underway to assess the safety, efficacy, and tolerance of VCTX211, an allogeneic gene-edited stem cell (PEC211)-derived product (Karpov et al., 2023). VCTX211 exhibits similar modality with that of VCTX210A, but the PEC211 consists of *B2M*, *TXNIP* deletion and *PD-L1*, *HLA-E*, *TNFAIP3*, *MANF* insertion to improve functionality and enhanced cell fitness in patients with T1D.

## Metabolic Disorder

**Calcific aortic valve stenosis:** Calcific aortic stenosis (AS) is a progressive fibrocalcific condition characterized by the gradual thickening and accumulation of calcium in the aortic valve leaflets. Over time, this process leads to severe narrowing of the valve, resulting in left ventricular hypertrophy and obstruction of cardiac outflow, which significantly limits blood supply to the body (Rajamannan et al., 2011). The pathogenesis of calcific AS is complex, beginning with fibrocalcific processes in the aortic valve, including the overproduction and disorganization of collagen fibers. These alterations are exacerbated by endothelial cell damage driven by lipid-derived species, cytokines, and other stressors, such as mechanical strain and radiation injury. Subsequently, LDL and lipoprotein(a) (LP(a)) infiltrate the valve, promoting the recruitment of inflammatory cells and accelerating inflammation and mineralization of the valve leaflets (Lindman et al., 2016).

Currently, no effective pharmacotherapy is available for this condition, and patients with severe cases require surgical aortic valve replacement. Due to the complex etiology of the disease, therapeutic efforts have focused on addressing contributory factors rather than the root cause. CTX-320, developed by CRISPR Therapeutics, targets *LPA* to reduce LP(a) expression by delivering Cas9 mRNA and sgRNA to the liver via LNP-mediated intravenous injection (Morrow et al., 2023). Preclinical data from cynomolgus monkeys demonstrated dose-dependent gene editing results, with a single 2 mg/kg infusion resulting in a 94% reduction in mean plasma LP(a) levels, which persisted until day 224. CTX-320 is currently undergoing a Phase I clinical trial (ACTRN12623001095651p) (CRISPR Therapeutics, 2023b).

**Hypercholesterolemia:** Patients with familial hypercholesterolemia (FH) are unable to recycle low-density lipoprotein cholesterol (LDL-C). Normally, LDL-C levels increase with age; however, patients are born with high LDL-C levels, resulting in plaque buildup and a high risk of coronary heart disease. FH is mostly caused due to the mutation of the *LDLR* (~80%) for LDL-C receptor (LDLR) that transports LDL-C from the blood into cells to use or remove from the body (Defesche et al., 2017). Additionally, mutations in *APOB* and *PCSK9*, which encode apolipoprotein B and proprotein convertase subtilisin/kexin type 9 (PCSK9), respectively, are responsible for FH (Alves et al., 2014; Cunningham et al., 2007). As the main component of LDL, apolipoprotein B plays an essential role in the interaction between LDL and LDLR (Behbodikhah et al., 2021). In the case of PCSK9, which regulates LDL receptor degradation via lysosomes, gain-of-function mutations decrease LDL transport to the liver, contributing to FH development (Abifadel et al., 2009).

VERVE-101 and VERVE-102, the therapeutic candidates for heterozygous FH (HeFH) developed by Verve Therapeutics, are designed to disrupt *PCSK9* in the liver by targeting the splice donor site to introduce a premature stop codon and inactivate *PCSK9* (Lee et al., 2023; Vafai et al., 2024). They utilize ABE mRNA and sgRNA to target *PCSK9*, which are encapsulated in standard LNP for VERVE-101 and proprietary GalNAc LNP in VERVE-102 (NCT05398029, NCT06164730) (Verve Therapeutics, 2022, 2024a). GalNAc LNP utilizes the GalNAc ligand, which binds to the asialoglycoprotein receptor (ASGPR) that is primarily expressed in the liver (Kasiewicz et al., 2023), whereas the standard LNP system mediates LDLR for endocytosis. This renders GalNAc LNP a more applicable liver-directed delivery system for patients with FH who have reduced LDLR levels. VERVE-101 or 102 was administrated by one-time intravenous infusion, and participants who received VERVE-101 showed a ~48% reduction in LDL-C and ~84% decrease in PCSK9 levels. However, the clinical trial of VERVE-101 was stopped because of side effects; the participant showed signs of organ damage and blood clotting. Participant recruitment is underway for VERVE-102; nonetheless, non-human primate (NHP) data have demonstrated a 62% decrease in LDL-C levels sustained for 6 months with a single infusion.

YOLT-101, developed by YolTech Therapeutics (NCT06461702) (YolTech Therapeutics, 2024b), attempts long-term inhibition of PCSK9 in patients with HeFH using the hpABE5 system in combination with optimized GalNAc LNP. Following administration to NHPs, YOLT-101 demonstrated sustained LDL-C reduction for up to 2 years. In a clinical study, patients who received 0.6 mg/kg dose of YOLT-101 exhibited 72.5% reduction in PCSK9 levels and 50.4% reduction in LDL-C levels at 16 weeks post-treatment as reported in a recent preprint (Wan et al., 2025).

Patients with refractory hypercholesterolemia (RH), including those with homozygous FH (HoFH) and compound-HeFH carrying two mutant *LDLR* alleles, result in at least two-fold higher plasma LDL-C levels than patients with HeFH. These individuals respond poorly to existing treatments, even at the maximal tolerable doses, and develop heart disease in the first two decades of life (Li and Wu, 2022). For instance, patients with HoFH are unresponsive to PCSK9-targeting drugs because they have a nonfunctional LDLR. To address this issue, VERVE-201 is currently in Phase Ib trial (NCT06451770) (Verve Therapeutics, 2024b). VERVE-201, comprising an mRNA encoding ABE and an sgRNA targeting the angiopoietin-like 3 (*ANGPTL3*) gene, was intravenously administered to reach the liver using GalNAc LNP (Lee et al., 2024). This drug was designed to inhibit ANGPTL3 expression in the liver to decrease the synthesis of LDL-C and triglycerides. Preclinical data for NHP infused with 3 mg/kg VERVE-201 presented a 95% mean reduction in blood ANGPTL3 levels, and in the HoFH NHP model, LDL-C levels decreased 46% from 458 to 247 mg/dL.

**Primary hyperoxaluria type 1 (PH1):** The inherited metabolic disorder primary hyperoxaluria type 1 (PH1) is due to mutations in the *AGXT* gene that encodes the hepatic enzyme alanine-glyoxylate aminotransferase (AGXT) (Latta and Brodehl, 1990), and loss or dysfunction of this protein leads to excessive oxalate production. Because oxalate cannot be further metabolized, it accumulates and is excreted in the urine, which results in progressive renal deposition and systemic oxalosis (Cochat and Rumsby, 2013).

YOLT-203 utilizes LNP delivery of a CRISPR-Cas12 system to permanently reduce oxalate levels by disrupting glycolate oxidase (GO), an enzyme encoded by the *HAO1* gene and critical for hepatic oxalate biosynthesis. Targeting *HAO1* rather than *AGXT* may offer therapeutic advantages, as PH1 can result from a heterogeneous spectrum of *AGXT* mutations (Williams et al., 2009). In a Phase I clinical study (NCT06511349) (YolTech Therapeutics, 2024a), the agent demonstrated the potential to normalize urinary oxalate excretion in patients with PH1; individuals who received the higher dose of 0.45 mg/kg exhibited a ~70% reduction in 24 h urinary oxalate levels, which was sustained throughout the 16-week primary observation period.

## Protein-folding Disease: Transthyretin Amyloidosis (ATTR)

Amyloidosis is caused by the accumulation of misfolded proteins in organs. Accumulation of misfolded transthyretin (TTR) protein in the body, usually in the heart, leads to transthyretin amyloid-cardiomyopathy (ATTR-CM), which makes the heart to thicken and become stiff (Ruberg et al., 2019). ATTR is classified into two types: wild-type ATTR (wtATTR), which is not associated with genetic mutations in the *TTR*, and hereditary ATTR (hAATR), which is associated with mutations in the *TTR*. Misfolding of TTR is related to the pH or temperature around the protein, and misfolding of wild-type TTR is related to aging, wherein the protein tetramer is damaged and dissociated into a monomer that becomes denatured and misfolded. In hATTR, the mutant *TTR* associated with ATTR-related symptoms is inherited from the parents and can manifest at any age (Kelly et al., 1997). In both cases, the median survival time after the disease onset without treatment is 2.5–3.5 years.

Research on the gene editing strategy has focused on knocking out the *TTR* in the liver because more than 99% of circulating TTR is produced in the liver. Based on this mechanism, clinical trials for ATTR-CM and ATTR-PN (polyneuropathy) using a drug named NTLA-2001, developed by Intellia Therapeutics, have progressed to Phase III (NCT04601051, NCT05697861, NCT06128629, and NC06672237) (Gillmore et al., 2021; Intellia Therapeutics, 2020, 2023a, 2023b, 2024b). NTLA-2001 incorporates human codon-optimized SpCas9 mRNA and sgRNA, both encapsulated within a liver-targeting LNP delivery system, and is administered via intravenous infusion. Proprietary LNP employs a ionizable lipid optimized for hepatic delivery, and enhance endosomal escape. A single administration of NTLA-2001 resulted in sustained reduction in serum TTR protein levels, observed as early as 14 days post-treatment, with patients receiving a 0.1 mg/kg dose demonstrating a reduction exceeding 47%, whereas those receiving 0.3 mg/kg achieved a reduction of more than 80%.

## Inflammatory Disease: Hereditary Angioedema (HAE)

HAE is a rare autosomal dominant disorder caused by mutations in the *SERPING1*, which encodes a C1 esterase inhibitor (C1-INH) that regulates contact activation pathways. Type I HAE arises from a deficiency in C1-INH, whereas Type II HAE results from dysfunctional C1-INH. Both forms lead to increased levels of bradykinin, a peptide that promotes vascular permeability and tissue swelling (Kaplan and Joseph, 2010).

The pathogenesis of HAE involves a cascade in which the *KLKB1* produces prekallikrein, which is indirectly activated by C1-INH into kallikrein. Subsequently, kallikrein acts on kininogen, leading to the generation of bradykinin, and which process is inhibited by C1-INH. Elevated bradykinin levels activate the bradykinin receptors 1 and 2, among which bradykinin receptor 2 is closely associated with the hallmark symptoms of HAE. These symptoms include episodes of severe and unpredictable swelling that can occur every few days or weeks. Swelling can last for several hours to days and can be life-threatening, particularly when it affects the throat.

NTLA-2002, developed by Intellia Therapeutics, targets *KLKB1* and blocks plasma kallikrein production, thereby preventing bradykinin formation (NCT05120830, NCT06262399, and NCT06634420) (Intellia Therapeutics, 2021, 2024a, 2025). This therapy comprises SpCas9 mRNA and sgRNA with liver-targeting LNP, which are administered as a single intravenous infusion to patients with HAE. Plasma kallikrein levels were reported to be maximally reduced by 8 weeks, with a 67% reduction in patients receiving 25 mg and a 95% reduction in those receiving 75 mg dose. Reduced kallikrein levels were sustained for at least 32 weeks in the 75 mg cohort and for more than 48 weeks in the 25 mg cohort. In terms of HAE attacks, patients in the study had baseline attack rates of 1.1 to 7.2 attacks per month. However, the 25 mg group demonstrated a 91% mean reduction in the attack frequency, and none of the patients in this group experienced any HAE attack 10 weeks after treatment (Longhurst et al., 2024).

## Cancers

The CRISPR-Cas9 system is applied not only to genetic disorders but also to acquired diseases, such as cancer, wherein the system is used to enhance lymphocytes and leukocytes to target and attack cancer cells. Recently, immunotherapy using chimeric antigen receptor-T (CAR-T), the genetically modified T cells expressing chimeric antigen receptors that recognize specific cancer cell antigens, has attracted attention because of its flexibility in targeting various cancer types.

In CAR-T and CAR-NK immunotherapies, CRISPR-Cas is employed to enhance the functionality and specificity of immune cells in targeting cancer cells. The most commonly used strategy is to knockout the *PD-1* in T cells, which binds to PD-L1 on the surface of cancer cells and suppresses T-cell activity (Zhao et al., 2018). Once eliminated, the modified T cells are activated to mount a strong immune response against cancer cells and reduce immune evasion of the cells (Ko, 2015; Wang et al., 2016). In this context, clinical trials of autologous tumor-infiltrating lymphocytes (TILs), knocked out for the *PD-1* using CRISPR-Cas9, are currently underway in patients with esophageal cancer (NCT03081715) (Hangzhou Cancer Hospital, 2017) and advanced hepatocellular carcinoma (HCC) (NCT04417764) (Central South University, 2019), as well as for PD-1 KO autologous EBV-specific cytotoxic T lymphocytes (CTLs) in patients with malignancies (NCT03044743) (Yang, 2017).

By knocking out the T-cell receptor (TCR) and PD-1 in CAR-T cells using the CRISPR-Cas system, cells can minimize the host immune response and respond more efficiently to their redirected targets. When applied to mesothelin-targeting CAR-T cells, which are in clinical trials in patients with mesothelin-positive multiple solid tumors (NCT03545815) (Chinese PLA General Hospital, 2018), this strategy can target mesothelin-overexpressing cancer cells while simultaneously addressing potential side effects, such as graft-versus-host disease (GVHD). Moreover, a clinical trial in patients with B-cell malignancies (NCT03166878) (Chinese PLA General Hospital, 2017) used a strategy that knocked out TCR and beta-2 microglobulin (β2m) to reduce MHC-I expression and improve immune recognition of CAR-T cells.

The NCT04502446 (CRISPR Therapeutics, 2020b) clinical trial employed a strategy to improve therapeutic efficacy while reducing the host immune response by simultaneously knocking out TCR and MHC-I in allogeneic CAR-T cells targeting CD70 overexpressed in specific cancer cells (Jacobs et al., 2015). In parallel, CAR gene insertion has been used to improve the functionality of CAR-T cells in an allogeneic environment. A similar strategy was used in NCT04244656 (CRISPR Therapeutics, 2020a), which aimed to improve the therapeutic efficacy and reduce the host immune responses by simultaneously knocking out TCR and MHC-I in CAR-T cells, targeting B-cell maturation antigen (BCMA), a protein highly expressed in multiple myeloma cells (Shah et al., 2020).

CD19 is a protein specifically expressed in B-cell carcinomas, and approaches that utilize CD19-targeting CAR-T cells while simultaneously silencing *PD-1* and *TCR* to increase persistence and anticancer activity are being explored. This strategy is also being investigated in a clinical trial (NCT04637763) (Caribou Biosciences, 2021) in patients with B-cell non-Hodgkin lymphoma. As a strategy to increase the effectiveness of the CAR-T cell therapy, a clinical trial (NCT04037566) (Xijing Hospital, 2019) is currently underway to improve the effectiveness of CAR-T cells targeting CD19 by inhibiting hematopoietic precursor kinase 1 (HPK1), a negative regulator that knocks down the TCR signaling pathway (Zhang et al., 2022b). An ongoing clinical trial (NCT04767308) (Huazhong University of Science and Technology, 2021) is aiming to further activate the T cell signaling pathway by turning off CD5, a negative regulator, along with TCR, allowing CAR-T cells to more effectively recognize and attack cancer cells. Furthermore, because CD5 has the potential to trigger fratricide between CAR-T cells, its removal may prevent self-destruction of CD5-targeted CAR-T cells.

Strategies to treat human papillomavirus (HPV)-related cervical cancer target the HPV E6 and E7 genes and virus-derived oncogenes that are expressed only in cancer cells. The E6 protein ubiquitinates and degrades the tumor suppressor protein p53 (Scheffner et al., 1990, 1993), an important protein that plays a role in cell cycle regulation, DNA damage repair, and apoptosis induction. When E6 inhibits p53, cells survive and proliferate in an abnormal state, making them more likely to become cancerous. The E7 protein binds to and inactivates another cancer suppressor protein, the retinoblastoma protein pRb (Giarrè et al., 2001). pRb plays an important role in cell cycle regulation by inhibiting the entry of cells from G1 to S phase. When pRb is inhibited by E7, the cell cycle progresses uncontrollably, resulting in abnormal cell proliferation. A clinical trial, NCT03057912 (First Affiliated Hospital, 2018), is underway to knock out E6 and E7 using a CRISPR-Cas9 plasmid surrounded by Poloxmer 407-based gel as a therapeutic strategy, administered twice per week for 4 weeks. Because E6 and E7 are only expressed in HPV-infected cells, treatment is likely to selectively induce apoptosis and growth inhibition in HPV-infected cervical cancer cells with no effect on normal cells (Honegger et al., 2015, Pal and Kundu, 2020).

## Viral Diseases

**Human immunodeficiency virus (HIV) infection:** The HIV, a member of the retrovirus family, specifically targets CD4^+^ T cells (Février et al., 2011). In the early stages of infection, there is a latency period that typically lasts approximately 10 years, when no new virions are produced; however, the viral genome integrates into the host cell DNA and remains intact. During the active phase of HIV infection, the virus rapidly replicates, and as the virus increasingly weakens the immune system and reduces the CD4^+^ T cell count to critical levels (below ~200 cells/μl), the person reaches the stage of AIDS (Krentz et al., 2004). Owing to the lifecycle of HIV, drugs that suppress HIV replication is ineffective against latent HIV infection (Siliciano et al., 2003). However, these latent cells may subsequently be reactivated, resulting in the production of new virions (Ruelas and Greene, 2013). Thus, latent HIV infection presents a major challenge to treatment.

Recently, clinical studies have been actively conducted on the use of CRISPR-Cas9 technology for HIV-1 treatment. For example, a clinical trial (NCT03164135) (Affiliated Hospital to Academy of Military Medical Sciences, 2017) demonstrated resistance to HIV in patients with hematological malignancies via *CCR5* knockout. In addition to HIV-binding to the CD4 receptor, the coreceptor CCR5 further facilitates cellular invasion (Bleul et al., 1997). In the trial, a patient with acute lymphoblastic leukemia showed remission 19 months after allogeneic stem cell transplantation from donor cells with a *CCR5* knockout without any gene editing-associated adverse effects. However, the proportion of modified lymphocytes was low (5%), prompting the exploration of advanced strategies to improve the efficiency of gene modification (Xu et al., 2019). Additionally, EBT-101, a CRISPR-Cas9-based gene therapy in Phase 1 clinical trials (NCT05144386) (Excision BioTherapeutics, 2022), is employing an AAV vector to deliver CRISPR-Cas9 and two sgRNAs to target flanking long terminal repeat 1 (LTR1) and GagD within the HIV-1 provirus by a single intravenous administration (Dash et al., 2019). Preliminary results from these trials on three participating patients have been published and revealed no dose-limiting toxicities or severe adverse events. A long-term follow-up study (EBT-101-002; NCT05143307) (Excision BioTherapeutics, 2023) is currently enrolling participants.

**Hepatitis B virus (HBV):** Chronic hepatitis B is a persistent inflammatory condition of the liver caused by infection with the hepatitis B virus (HBV) (Hoofnagle, 1990). The most widely used treatment related to nucleos(t)ide analogs (NA), which inhibit viral replication, along with immunomodulators that enhance the host immune responses (Zoulim and Locarnini, 2009). However, the covalently closed circular DNA (cccDNA) of HBV can persist within hepatocytes, presenting a major barrier to complete viral clearance and increasing the risk of viral reactivation upon discontinuing treatment (Richman, 2000). In addition, these therapies typically result in viral suppression rather than a functional cure, which is defined as the loss of the hepatitis B surface antigen (HBsAg) (Lai et al., 2007; Liaw et al., 2009). To address these limitations, TUNE-401 (NCT06671093) (Tune Therapeutics, 2024b) is being evaluated in an ongoing clinical trial. It utilizes mRNA encoding a sgRNA and dCas9 fused to a methyltransferase and an additional epigenetic repressor, delivered via LNPs through a single intravenous drip administration. The intervention targets conserved master controller sequence in HBVs to induce methylation of viral DNA and transcriptional repression that potentially facilitate sustained antiviral effects (Tune Therapeutics, 2024a).

## Primary Immunodeficiency Disease

Chronic granulomatous disease (CGD) is a rare inherited immunodeficiency disease that causes recurrent and life-threatening infections with bacteria, mycobacteria, and fungi (Heyworth et al., 2003). CGD results from mutations in genes encoding subunits of the phagocyte NADPH oxidase complex, which is responsible for generating ROS during the respiratory burst of phagocytosis. Affected neutrophils fail to produce sufficient ROS, leading to impaired microbial killing (Seger, 2010).

PM359 (NCT06559176) (Prime Medicine, 2024), developed by Prime Medicine, is an autologous HSPC therapy that represents the first clinical trial application of prime editing technology. It aimed to correct the delGT mutation in the *NCF1*, which causes the most common pathogenic variant of p47^phox^-deficient form of CGD. A single dose was well tolerated and restored NADPH oxidase activity to levels significantly exceeding the minimum threshold required for clinical benefit (Prime Medicine, 2025).

## Concluding Remarks and Future Perspectives

The CRISPR-Cas technology is an innovative approach for gene editing and is promising for the treatment of various genetic diseases. Unlike traditional treatments that only alleviate symptoms, this technology can target the root cause of both rare and common genetic disorders by directly and permanently modifying the causative genetic variants.

The therapeutic potential of this technology has led to extensive research collaboration. Universities and research institutions can leverage their advantages in basic research to provide enterprises with innovative technical approaches and theoretical support. In addition, large pharmaceutical companies are partnering with biotech firms specializing in the CRISPR-Cas technology to achieve complementary advantages. For example, the Broad Institute at MIT and Harvard University have collaborated with multiple biopharmaceutical companies to conduct research on CRISPR-Cas-based disease treatment, accelerating the translation of research into clinical applications. Similarly, Bayer and Editas Medicine reached a cooperative agreement to develop treatments for ophthalmic diseases based on the CRISPR-Cas technology.

Despite its transformative potential, several challenges remain in the therapeutic application of CRISPR-Cas technologies. Long-term safety risks include off-target effects, unintended mutations, and genotoxicity. Even with high-fidelity Cas variants and improved guide RNA design, rare but harmful off-target events cannot be ruled out. Moreover, the long-term consequences of genome editing, particularly in stem cells or tissues with proliferative capacity, remain poorly understood. Therefore, continuous preclinical and clinical monitoring, along with the development of reversible or self-limiting editing systems, is imperative. Another major challenge is immunogenicity. Pre-existing adaptive immune responses against Cas9, derived from bacteria, have been identified in humans. In particular, the major orthologs of the Cas9 protein, SpCas9 and SaCas9, have shown a high probability of stimulating human immune responses. Antibodies against SaCas9 and SpCas9 were detected in 78% and 58% of the donors, respectively, and antigen-specific T cells were identified in 78% and 67% of the donors, respectively (Charlesworth et al., 2019). These findings indicate that the potential immunological implications of the CRISPR-Cas9 system should be thoroughly evaluated for clinical application. Moreover, Ethical considerations mainly involve germline editing because somatic cell editing does not affect future generations. Although current clinical applications only focus on somatic cells, public concern is high following reports of embryo editing and highlighting the need for responsible use through transparency, public engagement, and strict oversight.

Owing to safety concerns, the FDA has conducted limited clinical trials on gene-editing technologies. Similarly, the World Health Organization has issued international guidelines emphasizing that gene-editing research must prioritize human safety and ethical considerations (World Health Organization, 2021). In 2023, Vertex Pharmaceuticals made significant progress in raising awareness with the approval of CASGEVY, a therapeutic agent that utilizes the CRISPR-Cas technology, through an ex vivo approach. This marks a significant milestone, suggesting its potential to meet safety and efficacy standards. To expand the use of the CRISPR-Cas technology in vivo, further research is required to address safety issues, optimize delivery methods, and improve the spatial configuration of the CRISPR-Cas systems. Efforts are being made to develop and improve various delivery systems, including LNPs and AAVs, with the need for new approaches to improve the stability and efficiency of gene-editing systems.

Despite the scope for improvement, the CRISPR-Cas technology is anticipated to become an essential tool for the treatment and management of various diseases for several reasons. First, with deeper research on gene functions and interactions, more precise gene editing can be achieved. It not only accurately corrects single-base mutations, but also adjusts complex gene structures, such as large fragment gene insertions, deletions, or replacements, while further reducing off-target effects to enhance treatment safety and effectiveness, thus enabling the effective treatment of more complex genetic diseases. Second, the development of more efficient and safe delivery systems can lead to more precise delivery of the CRISPR-Cas system to target cells, improve delivery efficiency, and minimize its impact on normal cells. For example, an LNP-based delivery system may be optimized in terms of structure and composition to better penetrate the cell membrane and deliver the CRISPR-Cas system into the cell nucleus. Third, personalized medicine will be readily available. Based on each individual’s unique genetic information, the CRISPR-Cas technology can be used to tailor personalized treatment plans. By precisely analyzing patient genes to determine specific mutation sites of pathogenic genes and designing targeted CRISPR-Cas gene-editing strategies, true precision medicine can be achieved, greatly improving treatment effects and reducing unnecessary side-effects. Finally, the application of the CRISPR-Cas technology will be expanded to include other fields. In addition to the currently focused areas, such as genetic diseases and cancer, the technology may provide breakthroughs in the treatment of infectious diseases, cardiovascular diseases, and metabolic diseases. As discussed in this review, as long as the CRISPR-Cas technologies continue to develop, they are expected to achieve remarkable breakthroughs in clinical application in the future, playing a pivotal role in biomedical sciences.

## Figures and Tables

**Table 1.** Delivery systems for CRISPR/Cas cargos

	Electroporation	AAV	LNP	AuNP
CRISPR/Cas Format	DNA, mRNA, RNP	DNA	DNA, mRNA, RNP	RNP
Advantage	High efficiency, versatility	High efficiency, specific tissue targeting	High efficiency, versatility	High efficiency, non-toxic, specific tissue targeting
Disadvantage	Cell toxicity, limited applicability	Capacity limitations, high production costs, time-consuming production	Limited tissue specificity long-term safety concerns, dose-dependent toxicity	Lower efficiency, complex manufacturing

**Table 2.** Clinical trials using the CRISPR-Cas technology

Disease	Target gene	Therapeutic approach	Editor	Delivery strategy	Product name	Sponsor	NCT ID	Phase
**β-Hemoglobinopathies**								
SCD/TDT	*BCL11A* enhancer	Gene disruption of the *BCL11A* erythroid enhancer in HSPCs via NHEJ	CRISPR-Cas9 RNP	Electroporation / ex vivo	CTX001 Exa-cel (Casgavy)	Vertex Pharmaceuticals & CRISPR Therapeutics	NCT03745287	Approved
							NCT05329649	
							NCT05951205	
							NCT03728322	
							NCT03655678	
							NCT05356195	
TDT	*BCL11A* enhancer	Gene disruption of the *BCL11A* erythroid enhancer in HSPCs via NHEJ	CRISPR-Cas9 RNP	Electroporation / ex vivo	BRL-101	Bioray Laboratories	NCT04211480	Phase I
							NCT04205435	
							NCT05577312	
							NCT06300723	
TDT	*BCL11A* enhancer	Gene disruption of the *BCL11A* erythroid enhancer in HSPCs via NHEJ	CRISPR-Cas9 mRNA and sgRNA	Electroporation / ex vivo	ET-01	EdiGene	NCT04925206	Phase I
							NCT04390971	
							NCT05752123	
TDT	*HBG1/2* promoter	Gene disruption of the binding sites of the *HBG1/2* promoter repressor in HSPCs via NHEJ	CRISPR-Cas9	Undisclosed / ex vivo	RM-001	Guangzhou Reforgene Medicine	ChiCTR2100052858	Phase I
							ChiCTR2100053406	
SCD/TDT	*HBG1/2* promoter	Gene disruption of the binding sites of the *HBG1/2* promoter repressor in HSPCs via NHEJ	CRISPR-AsCas12a RNP	Electroporation / ex vivo	EDIT-301 (Reni-cel)	Editas Medicine	NCT04853576	Phase I/II
							NCT05444894	
TDT	*HBG1/2* promoter	Gene disruption of the binding sites of the *HBG1/2* promoter repressor in HSPCs via NHEJ	CRISPR-Cas12b	Undisclosed / ex vivo	VGB-Ex01	Shanghai Vitalgen BioPharma	NCT06041620	N/A
SCD	*HBG1/2* promoter	Gene disruption of the binding site of the *HBG1/2 promoter repressor* in HSPCs via base editing	ABE	Electroporation / ex vivo	BEAM-101	Beam Therapeutics	NCT05456880	Phase I/II
SCD	*HBB*	Gene correction of the β-globin locus in HSPCs via HDR	CRISPR-Cas9 RNP with DNA template (AAV6)	Electroporation / ex vivo	KMAU-001	Kamau Therapeutics	NCT04819841	Phase I/II
					GPH-101	Graphite Bio		
					Nula-Cel			
SCD	*HBB*	Gene correction of the β-globin locus in HSPCs via HDR	CRISPR-Cas9 RNP with ssODN	Electroporation / ex vivo	CRISPR_SCD001	Mark Walters, MD	NCT04774536	Phase I/II
**Muscular diseases**								
DMD	*DMD*	*DMD* exon 50 skipping via base editing	CBE	AAV9 / in vivo	GEN6050X	Peking Union Medical College Hospital	NCT06392724	Early Phase I
DMD	*DMD*	Up-regulation of expression of the full-length isoform of dystrophin using a CRISPRa system consisting of dCas9 fused to VP64	CRISPRa	AAV9 / in vivo	CRD-TMH-001	Cure Rare Disease	NCT05514249	Phase I
DMD	*DMD*	*DMD* exon 51 skipping and restore the correct open reading frame	CRISPR-hfCas12Max	AAV / in vivo	HG302	HuidaGene Therapeutics	NCT06594094	Phase I
**Eye diseases**								
LCA	*CEP290*	Gene disruption of mutated allele in *CEP290* via NHEJ	CRISPR-Cas9	AAV5 / in vivo	EDIT-101	Editas Medicine	NCT03872479	Phase I/II
nAMD	*VEGF*	Knock down the expression of VEGFA	CRISPR-hfCas13Y	AAV / in vivo	HG202	HuidaGene Therapeutics	NCT06031727	Phase I
							NCT06623279	
**Auditory disorder**								
Congenital Hearing Loss	*OTOF*	RNA base editing of p.Q829X mutation in *OTOF* gene	CRISPR-Cas13	AAV / in vivo	HG205	HuidaGene Therapeutics	NCT06025032	Early Phase I
**Autoimmune diseases**								
T1D	knockouts (B2M, TXNIP) insertions (PD-L1, HLA-E, TNFAIP3, and MANF)	PEC210A (allogeneic pancreatic endoderm cells) or PEC211 (allogeneic stem cell) modified using CRISPR-Cas9	CRISPR-Cas9	Undisclosed / ex vivo	VCTX210A	CRISPR Therapeutics & Viacyte	NCT05210530	Phase I
					VCTX211		NCT05565248	Phase I/II
**Metabolic disorders**								
Calcific Aortic Valve Stenosis	*LPA*	Gene disruption of *LPA* via NHEJ	CRISPR-Cas9 mRNA and sgRNA	LNP / in vivo	CTX-320	CRISPR Therapeutics AG	ACTRN12623001095651p	Phase I
PH1	*HAO1*	Gene disruption of *HAO1* via NHEJ	CRISPR-Cas12	LNP / in vivo	YOLT-203	RenJi Hospital	NCT06511349	Early Phase I
**Hypercholesterolemia**								
HeFH	*PCSK9*	Gene disruption of* PCSK9* via base-editing	ABE mRNA and sgRNA	LNP / in vivo	VERVE-101	Verve Therapeutics	NCT05398029	Phase I
					VERVE-102		NCT06164730	
HeFH	*PCSK9*	Gene disruption of* PCSK9* via base-editing	ABE mRNA and sgRNA	LNP / in vivo	YOLT-101	YolTech Therapeutics	NCT06461702	Early Phase I
HoFH / RH	*ANGPTL3*	Gene disruption of *ANGPTL3* via base-editing	ABE mRNA and sgRNA	LNP / in vivo	VERVE-201	Verve Therapeutics	NCT06451770	Phase I
**Protein-folding disease**								
ATTR	*TTR*	Gene disruption of *TTR* via NHEJ	CRISPR-Cas9 mRNA and sgRNA	LNP / in vivo	NTLA-2001	Intellia Therapeutics	NCT04601051	Phase III
							NCT05697861	
							NCT06128629	
							NCT06672237	
**Inflammatory diseases**								
HAE	*KLKB1*	Gene disruption of *KLKB1* via NHEJ	CRISPR-Cas9 mRNA and sgRNA	LNP / in vivo	NTLA-2002	Intellia Therapeutics	NCT05120830	Phase III
							NCT06262399	
							NCT06634420	
**Cancers**								
Esophageal Cancer	*PD-1*	Gene disruption of *PD-1* in TILs via NHEJ	CRISPR-Cas9	Undisclosed / ex vivo		Hangzhou Cancer Hospital	NCT03081715	N/A
HCC	*PD-1*	Gene disruption of *PD-1* in TILs via NHEJ	CRISPR-Cas9	Undisclosed / ex vivo		Central South University	NCT04417764	Phase I
Malignancies	*PD-1*	Gene disruption of *PD-1* in EBV-specific CTLs via NHEJ	CRISPR-Cas9	Undisclosed / ex vivo		Yang Yang	NCT03044743	Phase I/II
Mesothelin-positive Multiple Solid Tumors	*PD-1* and *TCR*	Gene disruption of *PD-1* and *TCR* in anti-mesothelin CAR-T cells via NHEJ	CRISPR-Cas9	Undisclosed / ex vivo		Chinese PLA General Hospital	NCT03545815	Phase I
B-cell Malignancies	*B2M* and *TCR*	Gene disruption of *B2M* and *TCR* in anti-CD19 CAR-T cells via NHEJ	CRISPR-Cas9	Electroporation / ex vivo	UCART019	Chinese PLA General Hospital	NCT03166878	Phase I/II
Relapsed or Refractory T or B-Cell Malignancies	*B2M* and *TCR*	Gene disruption of *B2M* and *TCR* in anti-CD70 CAR-T cells via NHEJ	CRISPR-Cas9	Undisclosed / ex vivo	CTX130	CRISPR Therapeutics AG	NCT04502446	Phase I
Relapsed or Refractory Multiple Myeloma	*B2M* and *TCR*	Gene disruption of *B2M* and *TCR* in anti-BCMA CAR-T cells via NHEJ	CRISPR-Cas9	Undisclosed / ex vivo	CTX120	CRISPR Therapeutics AG	NCT04244656	Phase I
Relapsed or Refractory B-Cell Non-Hodgkin Lymphoma	*PD-1* and *TCR*	Gene disruption of *PD-1* and *TCR* in anti-CD19 CAR-T cells via NHEJ	CRISPR-Cas9	Undisclosed / ex vivo	CB-010	Caribou Biosciences	NCT04637763	Phase I
Relapsed or Refractory CD19+ Leukemia or Lymphoma	*HPK1*	Gene disruption of *HPK1* in anti-CD19 CAR-T cells via NHEJ	CRISPR-Cas9	Undisclosed / ex vivo	XYF19	Xijing Hospital	NCT04037566	Phase I
Relapsed/​Refractory CD5+ Hematopoietic Malignancies	*CD5 *and* TCR*	Gene disruption of *CD5* and *TCR* in anti-CD5 CAR-T cells via NHEJ	CRISPR-Cas9	Undisclosed / ex vivo	CT125A	Huazhong University of Science and Technology	NCT04767308	Early Phase I
**Viral diseases**								
HPV	HPV *E6*/*E7*	Gene disruption of HPV *E6*/*E7* via NHEJ	CRISPR-Cas9	LNP / in vivo		First Affiliated Hospital, Sun Yat-Sen University	NCT03057912	Phase I
HIV-1	*CCR5*	Gene disruption of *CCR5* in allogeneic stem cell via NHEJ	CRISPR-Cas9	Undisclosed / ex vivo		Affiliated Hospital to Academy of Military Medical Sciences	NCT03164135	N/A
HIV-1	5′- & 3′-LTRs and* gag*	Excising large portions of the HIV genome via NHEJ	CRISPR-Cas9	AAV9 / in vivo	EBT-101	Excision BioTherapeutics	NCT05144386	Phase I
							NCT05143307	
HBV	*HBV*	Epigenetic gene silencing through DNA methylation and heterochromatin formation	Epigenetic editing	LNP / in vivo	Tune-401	Tune Therapeutics	NCT06671093	Phase I
**Primary immunodeficiency disease**								
CGD	*NCF1*	Prime editing of HSPCs targeting *NCF1* mutation	PE	Undisclosed / ex vivo	PM359	Prime Medicine	NCT06559176	Phase I/II
